# Figure–ground discrimination behavior in *Drosophila*. II. Visual influences on head movement behavior

**DOI:** 10.1242/jeb.080192

**Published:** 2014-02-15

**Authors:** Jessica L. Fox, Mark A. Frye

**Affiliations:** Howard Hughes Medical Institute and Department of Integrative Biology and Physiology, University of California Los Angeles, Los Angeles, CA 90095-7239, USA

**Keywords:** Fly vision, Gaze control, Figure tracking, Optomotor response

## Abstract

Visual identification of small moving targets is a challenge for all moving animals. Their own motion generates displacement of the visual surroundings, inducing wide-field optic flow across the retina. Wide-field optic flow is used to sense perturbations in the flight course. Both ego-motion and corrective optomotor responses confound any attempt to track a salient target moving independently of the visual surroundings. What are the strategies that flying animals use to discriminate small-field figure motion from superimposed wide-field background motion? We examined how fruit flies adjust their gaze in response to a compound visual stimulus comprising a small moving figure against an independently moving wide-field ground, which they do by re-orienting their head or their flight trajectory. We found that fixing the head in place impairs object fixation in the presence of ground motion, and that head movements are necessary for stabilizing wing steering responses to wide-field ground motion when a figure is present. When a figure is moving relative to a moving ground, wing steering responses follow components of both the figure and ground trajectories, but head movements follow only the ground motion. To our knowledge, this is the first demonstration that wing responses can be uncoupled from head responses and that the two follow distinct trajectories in the case of simultaneous figure and ground motion. These results suggest that whereas figure tracking by wing kinematics is independent of head movements, head movements are important for stabilizing ground motion during active figure tracking.

## INTRODUCTION

Animals in motion generate large amounts of optic flow. Throughout the animal kingdom, perturbations to optic flow induce an optokinetic reflex in which the animal will attempt to minimize the perceived slip of the retinal image by compensatory head, eye or body movements (Paulus et al., 1984; Lappe et al., 1999). In vertebrates, rotations of the eyes allow the animal to stabilize retinal slip (Steinman and Collewijn, 1980; Miles, 1997). In an analogous response, flying and walking insects produce optomotor adjustments of their wing kinematics to rotate their entire body and similarly compensate for retinal slip (Hassenstein and Reichardt, 1956; Götz and Wenking, 1973; Götz, 1975). Similarly, they may show stabilizing responses by independently orienting their gaze by moving their heads (and therefore their eyes, as the eyes are fixed to the head). Previous studies of head movements in free-flying blowflies (Schilstra and van Hateren, 1998) and tethered flying *Drosophila* (Duistermars et al., 2012) showed that head movements are tightly coupled to wing steering kinematics during high-velocity body rotations: the head turns in the same direction as the thorax with a small delay and slightly faster kinematics. In blowflies, both the body and the head are stabilized during straight flight, and are tightly coordinated during saccadic turns such that head turns occur slightly later and faster than body turns, thereby minimizing the duration of motion blur (van Hateren and Schilstra, 1999). Head movements are used to stabilize gaze and minimize motion blur during body rotations in roll, pitch (Hengstenberg, 1991) and yaw (Land, 1973) in tethered flies.

In addition to the optomotor responses that stabilize wide-field panoramic motion, *Drosophila* also orient toward small contrasting figures both while walking (Schuster et al., 2002; Robie et al., 2010) and in tethered flight (Götz, 1975). In both *Drosophila* and houseflies, figure fixation in flight can be evoked simply with a high-contrast vertical bar (Götz, 1975; Reichardt and Poggio, 1976). Orientation responses toward small figures and stabilizing responses to wide-field perturbations differ in their sensitivity to stimulus size and their dynamics (Egelhaaf et al., 1988; Duistermars et al., 2007). Little is known about the role of head positioning in figure tracking, and, in particular, figure tracking against a moving wide-field background. How might flies stabilize their gaze when tracking a small-field moving figure while simultaneously stabilizing a wide-field ground, as would generally occur during natural figure-tracking flight behavior? Do flies attempt to fixate the object with their gaze (object fixation behavior), do they use gaze to reduce wide-field retinal slip (optomotor stabilization behavior) or do they exhibit some composite response to both stimuli?

To determine how *Drosophila melanogaster* Meigen 1830 stabilizes its gaze when tracking figure motion against ground motion, we measured head and wing movements of tethered flies during presentation of stimuli that could be distinguished only by relative motion. We used linear systems analysis techniques to measure spatiotemporal action fields (STAFs), representing the spatial variation of the input–output function of optomotor responses, for both wing-steering and head-angle responses. This linear systems approach provides a good approximation of the overall input–output relationship between wide-field and figure motion wing-steering responses (Theobald et al., 2010; Aptekar et al., 2012). The robust linearity demonstrated by these analyses does not imply that the underlying mechanisms are linear, but rather that the many inherent nonlinear processes combine – consistent with the central limit theorem – to produce linear responses over the performance operating range. Here, we used this approach to examine wing-steering and head-movement behaviors to both figures and wide-field motion simultaneously. STAFs are

**List of abbreviations**

FD: figure-detecting
HS: horizontal system
IR: infrared
LPTC: lobula plate tangential cell
m-sequence: maximum length sequence
STAF: spatiotemporal action field
VS: vertical system
ΔWBA: difference between left and right wingbeat amplitudes

qualitatively similar to the spatiotemporal receptive fields used to describe responses of single neurons to stimuli varying in space and time (DeAngelis et al., 1999), but they describe the animal's behavioral response rather than neural activity. By describing amplitudes, time courses and spatial profiles of responses of both heads and wings to visual stimuli, we are able to investigate the ways in which the fly responds with independent wing steering and gaze shifts in response to compound visual stimuli. In particular, we are able to quantify the degree to which the flies are exhibiting small-field object (figure) fixation or wide-field optomotor ground stabilization with their wing steering and head movements.

We find that *D. melanogaster* uses its head to stabilize ground motion and does not attempt to track figures with gaze when ground motion is present, which is distinct from its wing-steering behavior. Our results suggest that there are multiple strategies for gaze control employed by flying insects, and that these strategies could be influenced by multiple sensory inputs or a particular behavioral task. Furthermore, the contrast of our results with other studies of gaze control in insects indicates that these strategies may vary significantly between species.

## RESULTS

### Head movements are required for figure tracking against wide-field motion in closed-loop conditions

**Fig. 1.**
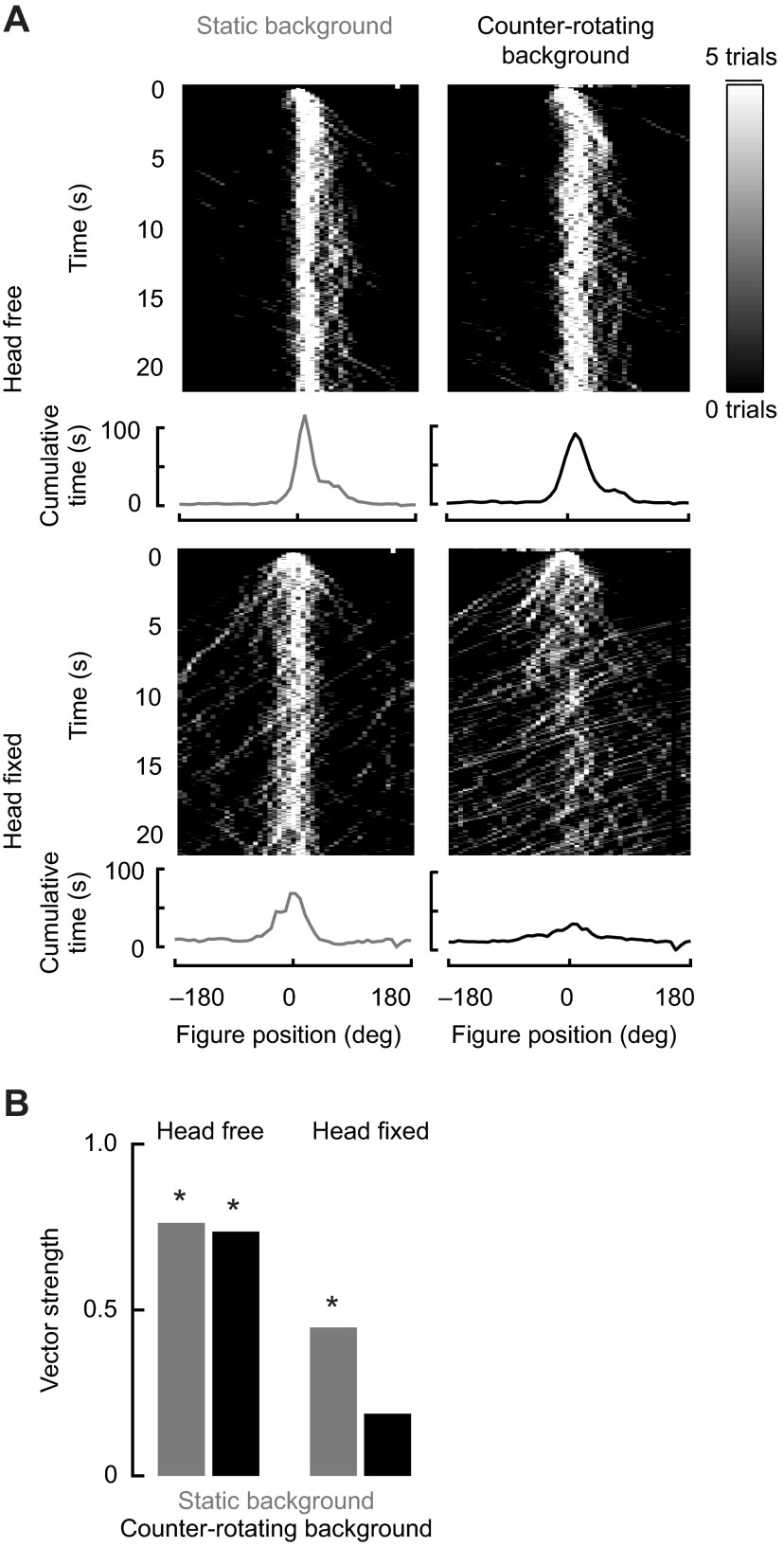
**Figure tracking under closed-loop control with and without head movements.** In these experiments, flies were able to control the position of the figure stimulus by steering their wings. (A) Traces of figure position during all closed-loop trials. Top row: traces for head-free flies (*N*=11 flies, 55 trials); bottom row: traces for head-fixed flies (*N*=12 flies, 60 trials). Left: moving figure on static wide-field panorama; right: moving figure on counter-rotating wide-field panorama. Graphs underneath each set of traces represent the position of the bar during the last 15 s of each trial. (B) Vector strength measurements for the last 10 s of each trial. Asterisks indicate where flies are fixating the figure, as determined by a Rayleigh *z*-test (*P<0.05). Head-free flies are able to fixate the figure regardless of wide-field motion; head-fixed flies are able to fixate the figure on a static wide-field panorama, but not a counter-rotating wide-field panorama.

We first measured the fly's ability to actively track a moving randomly textured figure set against a similarly randomly textured ground with their heads free to move (Fig. 1A, top row), and with their heads fixed in place with a drop of glue (Fig. 1A, bottom row). Flies were provided with active closed-loop control over the moving figure by means of inversely coupling the difference in WBA to the rotational velocity of the image. When the figure was moving on a stationary ground, both groups of flies were able to robustly stabilize the figure in the frontal field of view, which results in a distinct peak in the probability density function of the figure's position at 0 deg azimuth (Fig. 1A, left). We quantified frontal fixation by calculating vector strength, a circular statistic that measures the degree of similarity in a set of angular measurements. This calculation allowed us to determine whether the distribution of figure positions was significantly different from a uniform distribution around the arena at the *P*<0.05 level using a Rayleigh *z*-test (Batschelet, 1981). For this experiment, the *z*-test indicates whether the population of flies is achieving statistically significant fixation (n.b. it is not a comparison across experimental treatments). For a static-panorama condition, head fixation resulted in only a slight decrease in vector strength, and head-fixed flies passed the *z*-test by strongly tracking the figure against the static panorama (Fig. 1A, bottom left). We then challenged the flies by programming the ground stimulus to counter-rotate equally for any displacement of the moving figure, such that any steering attempt by the fly to bring the figure toward the midline resulted in a ground displacement in the opposite direction at the same speed. Under these conditions, we might expect that the flies' optomotor response would result in an attempt to stabilize the

ground motion and thus hinder their ability to frontally fixate the figure. However, head-free flies were able to stabilize the figure nearly as well as they could against a static background. In contrast, flies with fixed heads could not fixate the figure (Fig. 1A, right). This demonstrates that head movements are necessary for actively tracking a moving figure superimposed upon a counter-rotating ground. This result motivated us to separate the responses to figure motion and ground motion by presenting them under controlled open-loop conditions.

### Fixing the fly's head decreases wing-steering responses to wide-field motion, but not figure motion

**Fig. 2.**
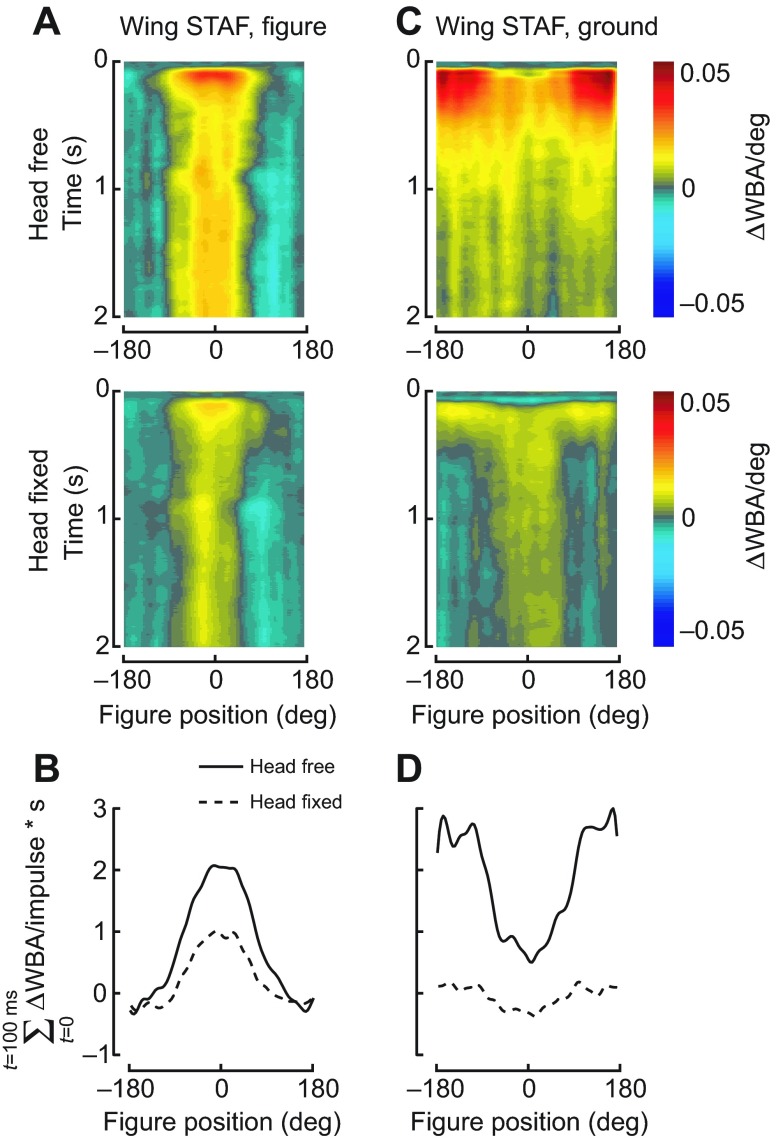
**Figure and wide-field spatiotemporal action fields (STAFs) for wild-type and head-fixed flies.** (A) Figure STAFs for head-free (top) and head-fixed (bottom) flies. (B) Integration of the figure STAF over the first 100 ms of the response for head-free and head-fixed flies. (C) Wide-field STAFs for head-free and head-fixed flies. (D) Integration of the wide-field STAF over the first 100 ms of the response. The wide-field response is diminished by fixing the head. Wild-type flies, *N*=27 [reprinted from Fox et al. (Fox et al., 2014)]; head-fixed flies, *N*=22. ΔWBA/deg, metric of the fly's steering response (difference between left and right wingbeat amplitudes) per degree of stimulus motion.

We measured the wing-steering STAFs to both moving figure and moving ground panoramas when the head was fixed in place, and compared these results with those from flies with freely moving heads [Fig. 2A,C, top row, re-plotted from Fox et al. (Fox et al., 2014)]. In flies with freely moving heads, the largest response to the figure occurs when it is in the frontal visual field, with little to no responses to the figure when it is displaced into the visual periphery (Fig. 2A). By contrast, responses to ground motion are large when the figure is in the periphery, and are attenuated when the figure is in the front of the field (Fig. 2C).

The strength of this analysis is that any changes to the way visual signals are transformed into motor responses would be reflected in the amplitude, time course or spatial structure of the STAFs, and examining these changes can be informative regarding the functioning of the system. Here, the ‘system’ refers to the entire cascade from visual input (wide-field optic flow or figure motion) to behavioral output (wing kinematics or head movement), and changes in any part of the cascade are reflected in the STAF.

How much of the structure of the wing-steering STAF is dependent on head movements, and what is the effect on the STAFs if the head movements are eliminated? We find that fixing the fly's head and modulating ground motion and figure motion with independent white-noise maximum length sequences (m-sequences) results in only slightly decreased responses to figure motion, and most of the observed difference occurs only within the early-onset component of the response (Fig. 2A, compare top and bottom). The figure STAFs are similar in amplitude during the latter part of the response, and integrating over the first 100 ms of the response shows that the two STAFs have similar spatial profiles, with a slight amplitude reduction centered on the visual midline and absent in the visual periphery, where there is no influence of head fixation at all (Fig. 2B). By contrast, the wide-field response is sharply decreased by head fixation, particularly when the figure is displaced within the visual periphery (Fig. 2C, bottom panel). The amplitude of the ground STAF for head-fixed flies nowhere approaches the peak amplitude of the STAF for head-free flies. Integrating over the first 100 ms of the response shows that both head-fixed and head-free wide-field STAFs have their highest amplitudes when the figure is in the rear of the visual field, and their lowest amplitudes when the figure is in the front (Fig. 2D). However, the large-amplitude responses to ground motion, superimposed with figure motion in the periphery, are severely decreased by head fixation; the small-amplitude response to ground motion superimposed with figure motion positioned frontally is only slightly diminished. The spatial structure of the ground STAF, with a decreased response when the figure is in the frontal field of view, is similar between head-fixed and head-free flies (Fig. 2D). This indicates that the overall response to ground motion is simply attenuated, and not disordered, by the removal of head movement input.

The figure and ground wing STAFs for head-fixed and head-free flies suggest that figure tracking is nearly independent of head movements, because steering responses to figure motion persist largely unaltered when head movements are restricted. This is entirely consistent with published reports of frontal bar fixation by *Drosophila*, which frequently use head-fixed flies so that the retinal position of visual stimuli is unambiguous (Tammero and Dickinson, 2002). By contrast to figure fixation responses by the wings, head movements are essential for the full-amplitude response to ground motion, particularly when the figure is in the periphery and the majority of the finite steering effort is directed toward wide-field stabilization.

### Wing steering follows a combination of ground and figure motion, whereas head movements are correlated with ground motion only

Given that head fixation dramatically alters wing-steering behavior in response to a compound visual stimulus containing figure and ground motion, we sought to directly measure head responses to these compound visual stimuli during flight. What are these head movements that are so essential to ground stabilization? Do head movements simply follow wing steering movements with a short delay, as they do in response to wide-field motion in other experiments (Schilstra and van Hateren, 1998; Duistermars et al., 2012),

**Fig. 3.**
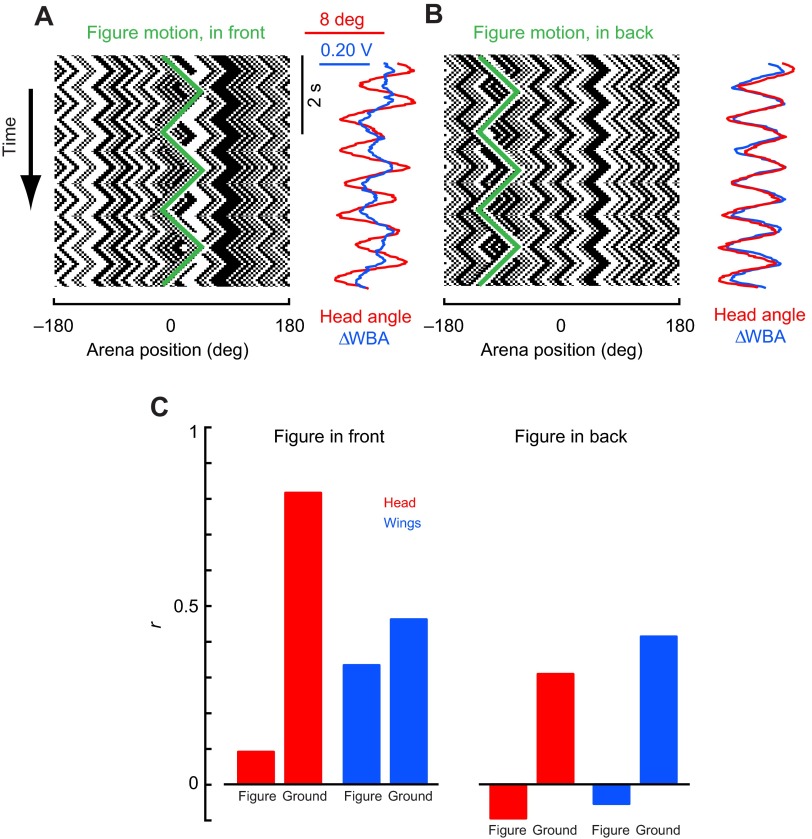
**Head and wing responses to triangle-wave figure and ground stimuli with the figure stimulus appearing in the front or the back of the fly's visual field.** (A) Space–time plot of the stimulus, with the figure on the visual midline, and average responses for figure motion in the front of the visual arena. Wing-steering responses represent a composite of the figure motion and the wide-field motion, while head angle responses are similar to wide-field motion only. (B) Space–time plot of the stimulus and average responses for figure motion in the rear of the visual arena. Here, both wing and head responses are similar to the wide-field motion. (C) Correlations between the wing-steering (blue) or head-movement (red) response and the motion of each part of the stimulus for figures in the front (left) or the back (right) of the visual field. *N*=15 flies, 150 trials of each experiment, figure motion 60 deg and ground motion 30 deg in total peak-to-peak amplitude.

or might they respond differently to a compound visual stimulus? We recorded head angle, along with wing kinematics, while flies viewed a stimulus consisting of figure–ground stimuli moving with simple periodic triangle-wave trajectories oscillating at two different frequencies.

When figure oscillation is centered on the visual midline, the wing optomotor steering response follows a compound trajectory, reflecting both the slow frequency of figure motion (at 0.5 Hz) and the faster frequency of ground motion (at 1.2 Hz; Fig. 3A, blue trace). When the figure is offset into the visual periphery, the steering effort is predominantly correlated with ground motion, with no apparent contribution from the figure motion signal (Fig. 3B, blue trace). This result is entirely consistent with the wing-steering STAFs: steering response kernels are largest when the figure is centered near the midline and decay when the figure is in the visual periphery. By contrast, optomotor steering responses to the ground motion are largest and fastest when the figure is displaced into the periphery.

If wing-steering responses are dependent on figure position, does the same hold true for head movements? Because head movements follow wing steering when flies experience wide-field optic flow, we expected that head movements would be tightly coupled to wing steering and would track a frontally located figure. Instead, we were surprised to find that, regardless of whether the figure is in the front (Fig. 3A) or the rear field of view (Fig. 3B), head dynamics closely follow ground motion with no apparent response to the figure. We measured the correlation between the motion of the two stimulus components and the fly's head and wing responses, and found that head movements are strongly correlated to ground motion when the figure is in the frontal visual field (Fig. 3C). This is strikingly different from the wing response to the same frontal figure, which is correlated to both figure and ground motion in nearly equal proportions (Fig. 3C). When the figure is in the rear of the visual field, both heads and wings are strongly correlated to ground motion (Fig. 3C).

**Fig. 4.**
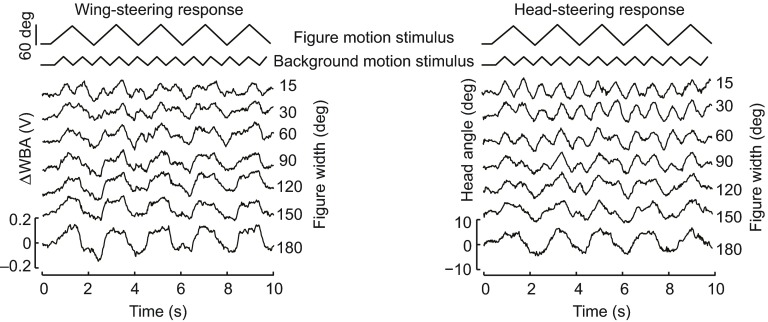
**Influence of varying figure width on head and wing responses to triangle-wave figure and ground stimuli.** For the same experiment described in Fig. 4, and only for the figure centered on midline, the horizontal width of the figure was systematically varied from 15 to 180 deg. Each plotted trajectory is the mean response for *N*=15 flies. Note that for even the smallest width figures, the wing-steering trajectories contain components of both the figure and ground stimuli, whereas the head trajectories follow only the ground component.

We repeated these triangle-wave experiments with figures of increasing width. For smaller figures (15 and 30 deg wide), the wing-steering responses are complex, reflecting components of both the figure and ground motion trajectories (Fig. 4). By contrast, and consistent with the results presented in Fig. 3, the head trajectories at these sizes show only the higher-frequency ground stimulus component (Fig. 4). For intermediate figure sizes between 60 and 180 deg, both the head and wing responses systematically show greater response to the low-frequency figure. For the maximum size figure we tested, occupying the frontal 180 deg of the arena, the wing and head trajectories are essentially indistinguishable, clearly following the motion of the low-frequency stimulus presented in the frontal half of the arena (Fig. 4).

### Head STAFs show that gaze is correlated with ground motion, but not figure motion

**Fig. 5.**
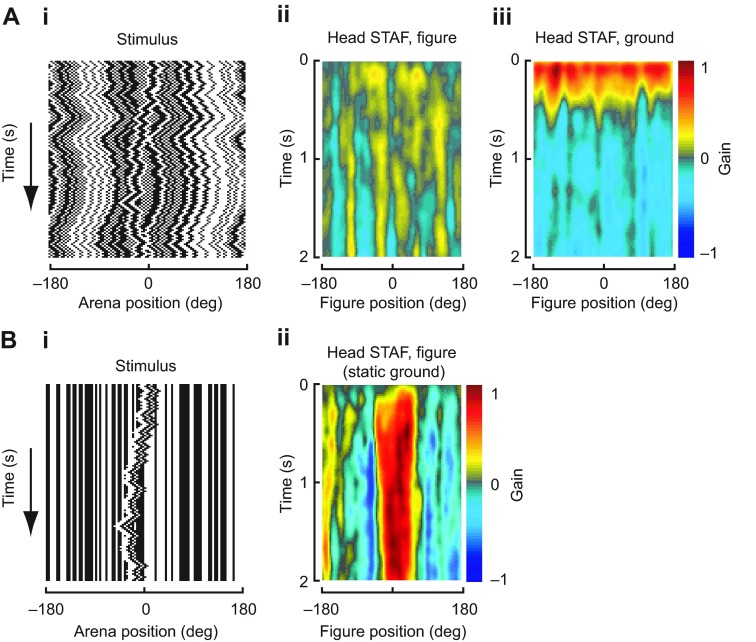
**Head-movement STAFs.** These STAFs are calculated using the same methods as the wing STAFs shown in Fig. 5, but show correlations between stimulus motion and head angle rather than correlations between stimulus motion and wing steering. (A) Figure and wide-field head STAFs. (i) Space–time plot of a sample stimulus used to construct the STAF, with figure and wide-field panorama moving according to two random sequences; (ii) head STAF of responses to figure motion; (iii) head STAF of responses to wide-field motion. In contrast to wing-steering figure and wide-field STAFs, the fly's responses are limited to the wide-field stimulus, with no response to the figure stimulus, and are uniform over the visual azimuth. *N*=25 flies. (B) Head STAF for a figure moving on a static wide-field panorama (*N*=13 flies). (i) Space–time plot of figure stimulus moving on a static wide-field panorama; (ii) head STAF of responses to figure motion. When the wide-field panorama is static, the head movements are correlated with figure motion while the figure is in the frontal field of view.

To fully examine the dynamics of the fly's head movements in response to figure and ground stimuli over the entire visual azimuth, we measured head STAFs by taking the cross-correlation of the head's movement with the figure or ground motion during trials

where the figure and ground moved according to independent white-noise sequences (Fig. 5Ai). This method is computationally identical to the technique used to measure wing-steering STAFs above, but finds the correlation between the stimulus and the head angle rather than the correlation between the stimulus and ΔWBA. In doing so, we measured the amplitude and spatiotemporal dynamics of the head's response to each stimulus component, for figure motion sampled across the visual azimuth.

We find that head responses to both figure and ground motions are spatially uniform, i.e. similar across the azimuth, with no discernible response to the moving figure (Fig. 5Aii) and strongly correlated responses to ground motion regardless of figure position (Fig. 5Aiii). This is in sharp contrast to the wing-steering STAFs (Fig. 2A), in which the figure and ground responses are mutually exclusive across the visual azimuth. The head movements are correlated to the ground motion regardless of the figure's position. Thus, when the figure is in the frontal field of view, the fly's wings are predominantly correlated with figure motion (Fig. 2A), but the head movements minimize retinal slip of the wide-field ground regardless of figure position (Fig. 5Aiii), further demonstrating the separability of the optomotor control of the wings and head.

Are there scenarios in which flies might track small moving figures with their heads, or is ground stimulation required for visually induced head movements? To answer this question, we measured STAFs using a moving figure against a static randomly textured wide-field panorama (Fig. 5Bi). When the ground is stationary, flies indeed follow figure motion with their heads (Fig. 5Bii). The situation in which both figure and ground are moving independently is the only case for which we find that the wings and heads follow distinct trajectories. This result confirms that flies can follow the motions of small figures with their gaze, but this response is completely overridden in the presence of wide-field ground motion.

## DISCUSSION

To examine gaze stabilization strategies for simultaneous figure and wide-field motion in *D. melanogaster*, we measured wing-steering and head-angle responses of tethered animals to moving figures set against independently moving wide-field grounds. We took a linear systems analysis approach and measured impulse responses with white noise stimuli, and also measured wing-steering and head-angle responses to periodic stimuli (Figs 3, 4). The head-movement and wing-steering motor systems behave approximately linearly over the operating range tested, and thus despite underlying neuronal non-linearities, a linear systems analysis is useful for both describing behavioral dynamics and spatial sensitivity of the system and identifying any pronounced nonlinearities. We found that head movements are necessary for robust figure tracking against a counter-rotating ground (Fig. 1) and for normal-amplitude wing-steering optomotor responses (Fig. 2). We were surprised to find that whereas wing-steering responses indicate spatially dependent compound figure and ground tracking (Fig. 2A, Fig. 3), head responses are strictly correlated to ground motion with no apparent influence of superposed figure motion (Fig. 3, Fig. 5A). When the ground is stationary, however, head movements, like wing movements, follow figure motion.

Freely flying blowflies turn their head and thorax (via the wings) in sequence and in the same direction in a general effort to maintain stable visual gaze when there is no moving figure present (Schilstra and van Hateren, 1998), and foraging dragonflies track a figure with similarly coupled head and body kinematics when capturing prey (Olberg et al., 2007). Ours is the first demonstration that actions of wings and heads during flight in flies are separable and are influenced differently by these two visual stimuli. Our results, in light of descriptions of head movements in other insects, indicate that head movements are gated by wide-field optic flow, and as such there exist parallel motor strategies for controlling gaze during wide-field ground stabilization and figure tracking during flight. These results also suggest possible neural mechanisms for rapidly and accurately adjusting gaze in flight.

### Intact head movements are required for proper wing kinematics during wide-field stabilization but not for figure tracking

Experiments in which we fixed the flies' heads show that if the heads are immobilized, then wing responses to wide-field ground motion are greatly reduced, particularly when a figure is in the visual periphery (Fig. 2C,D). However, the converse is not true: fixing the heads results in only a modest reduction in the amplitude of wing-steering responses to figure motion (Fig. 2A,B). In general, we observe that fixing the heads leads to a small systemic reduction in the strength of all wing optomotor responses, but a large reduction specifically in wide-field responses. In experiments with an identical flight arena, Reiser and Dickinson (Reiser and Dickinson, 2013) displayed a wide-field pattern of translatory optic flow and found that under some conditions, head-free flies preferentially steer toward the focus of visual expansion (reflecting normal forward flight) but fixed-head flies do not. Thus, both in their and our experiments, head fixation interferes with normal wing-steering optomotor behavior.

Our experiments furthermore highlight the role of head movement in the wing-steering control effort for optomotor stabilization, rather

than figure tracking. When wing steering is less correlated with the wide-field motion, as occurs when the figure is in the front of the visual field (Fig. 2A), the head movements are nonetheless strongly correlated with ground motion (Fig. 5Aiii). Additionally, flies do not attempt to track the figure with their heads when the ground is also moving (Fig. 5Aii). However, when the figure is expanded to 90 deg or more, the head then begins to track it as if it were a ground stimulus (Fig. 4). These results reveal distinct visuomotor strategies with which flies track ground and figure motion, and that compound figure–ground stimulation uncouples these distinct control efforts for head movements and wing kinematics, respectively. When figure motion is superposed upon ground motion (which would occur in any normal case of ego-motion), then the wing control effort is shared between figure and wide-field tracking, but the head control effort is dominated by wide-field gaze stabilization to the exclusion of figure tracking.

One potential benefit of reducing retinal slip with optokinetic head movements is that the relative motion of small figures will be enhanced, resulting in visual pop-out and making the figure more salient (Zahar et al., 2012). However, such optokinetic stabilizing responses of the head are not strictly required for figure tracking by the wings (Fig. 2A). Figure-tracking behavior may benefit from the increased figure motion salience that results from the stabilizing movements of the head, but our data indicate that stabilizing the background for motion pop-out is not strictly necessary for figure-tracking behavior.

### Potential involvement of efference copy in head movements

Head movements track a small figure superimposed on a stationary panorama (Fig. 5B), yet this response is superseded by the presence of wide-field ground motion (Fig. 5Aiii). Furthermore, figure tracking is not severely impaired by fixing the head (Fig. 1A), but figure tracking against ground motion is. This indicates that figure-tracking behavior does not require gaze stabilization or gaze pursuit per se, but rather only requires gaze stabilization to compensate for perturbations to wide-field optic flow superimposed on the moving figure. Such a bipartite system would preserve figure tracking against an unstable background, e.g. if the fly were displaced by a wind gust, thereby canceling the corrupting influence of motion blur on the motion salience of the figure. This stabilization strategy ensures that a fly is able to cope with perturbations, both self-generated and external, without losing track of its target.

Although tethered experiments can accurately simulate aspects of free flight, there are some fundamental constraints to such comparisons. Most notably, tethering flies under open-loop feedback conditions by definition removes the visual reafference (the sensory input associated with the animal's own action) that would occur with body rotations in free flight, as attempted steering results in no changes in the visual input. Thus, although the motor command from the wing-steering system is still generated by attempted turns and consequently any putative efference copy (an internal duplicate of the motor command) is intact, the expected change in the visual stimulus (expected reafference) is eliminated. In head-fixed open-loop experiments (Fig. 2), the expected reafference from both wing steering and head rotations is absent. How might this affect the fly's wing steering and head movements in response to figure and wide-field motion?

In vertebrates, efference copy is used during active pursuit eye rotations to suppress the optomotor nystagmus reflex that would otherwise keep the eyes locked to the moving panorama (Cullen, 2004). Similarly, previous work has found that an efference copy may be necessary to suppress the optomotor response to reafference and enable a fly to track objects (von Holst and Mittelstaedt, 1950; Chan et al., 1998); however, a physiological instantiation of visual efference copy has yet to be identified in flies. Could the lack of reafference in open-loop testing conditions spuriously generate the strong optomotor head movement response seen here? Several of our results suggest not. During closed-loop experiments, the animal can generate reafference by moving its head and steering its wings, and yet the results of these experiments are consistent with open-loop results in showing that flies require head movements to stabilize wide-field motion. Also, the motor command, and thus the efference copy, for wing steering is intact in all experiments, and thus it is unlikely that the optomotor fixation observed in head movements is artificial. By contrast, if the efference copy were necessary for object fixation and yet suppressed simply by tethering, then flies would be unable to follow figures in the open-loop condition, which they do with their head movements when the ground pattern is stationary (Fig. 5B). Finally, the dynamics of open-loop figure-tracking wing kinematics by flies tethered rigidly within this arena are virtually identical to the body kinematics of flies tethered magnetically and freely rotating about the yaw axis (Theobald et al., 2008). Taken together, our results consistently indicate that if efference copy mechanisms are at work in stabilizing superposed figure and ground motion, then such mechanisms are not being disabled by our experimental condition to the point of interfering with our interpretations.

In general, ‘inner-loop’ reflex behaviors such as the optomotor response are not informed by efference copies, whereas outer-loop goal-directed behaviors such as figure tracking result in an efference copy that can be used to modulate sensory input (Chan et al., 1998) or motor output (Viollet and Zeil, 2013). Efference copy can be useful in suppressing reflexes in favor of goal-directed behavior, for example suppressing the optomotor response in order to track a small figure. The wing-steering behavior exhibits this suppression (Fox et al., 2014), although our data do not indicate a particular neural mechanism for this behavior. However, the head movements do not show reflex. This may indicate that the head movements do not have access to the efference copy generated by the wing steering, which is in contrast to recent findings in hymenopteran insects (Viollet and Zeil, 2013). However, the crucial role of mechanosensation in dipteran head stabilization (Hengstenberg, 1991; Paulk and Gilbert, 2006; Huston and Krapp, 2009) and apparent lack of such input in hymenopteran head stabilization (Viollet and Zeil, 2013) indicate that there are multiple neural mechanisms at work in flying insects for gaze control. Future experiments will examine gaze control in flies during both inner-loop and outer-loop behaviors to determine the role that efference copy plays in each, an open question not addressed by the data presented here.

### Potential neural circuits for visual control of head movements

By contrast to a high-order mechanism by which motor commands are copied and subtracted from reafferent signals to enable figure–ground discrimination, simpler lower-order mechanisms are also at work. Flight equilibrium responses are mediated by slow visual and fast mechanosensory systems (Sherman and Dickinson, 2003). Indeed, cooperative rapid mechanosensory input via halteres strongly influences head movements in blowflies (Hengstenberg, 1991), likely because of the requirement for both visual and mechanosensory input for spiking activity in some neck motoneurons (Huston and Krapp, 2009). The neck motoneuron receptive fields, their multisensory gating and the flies' behavioral responses suggest that once there are sufficient mechanosensory inputs for the head to move, its trajectory is informed by the visual system (Hengstenberg, 1991). Our data suggest that during stimulation with both figure and wide-field motion, head movements rely exclusively on input from the motion–vision pathway, which is neurally distinct from the object–fixation pathway (Bahl et al., 2013). Nevertheless, the object–fixation pathway does inform head movements during stimulation with a figure only (Fig. 5B), indicating that the visual input to the gaze control mechanism is context dependent and may even require a switch between the two pathways.

The requisite visual signals arrive, in large part, from motion-collating neurons of the third optic ganglion, the lobula plate. Our results are consistent with the finding that wide-field motion is encoded by the neck motoneurons that drive head movements, and generalize this finding from the neural level to the behavioral level (Huston and Krapp, 2008). Our analysis indicates that the responses of neck motoneurons to wide-field optic flow, as described by Huston and Krapp (Huston and Krapp, 2008), ultimately result in strong tracking of wide-field motion by head movements. The visual inputs from horizontal system (HS) and vertical system (VS) lobula plate tangential cells (LPTCs) to neck motoneurons (Wertz et al., 2012) are the most likely source of the information needed to rotate the head in response to wide-field motion. We would suggest that a wide-field motion pathway that (1) shows high gain to small-field motion and (2) projects to and informs the neck motor system for yaw head kinematics, as does the HS class of LPTCs (Huston and Krapp, 2008; Lee and Nordström, 2012), would explain the apparent gating phenomenon we observe: that head kinematics follow figures when there is no wide-field motion [which could result from inputs of a cell that is high gain for small-field figures, in combination with a neck motoneuron with a small receptive field (Huston and Krapp, 2008)], but exclusively follow wide-field motion when it is active (resulting from the input of a cell that is optimally tuned for wide-field signals). Using HS input to drive head motions, the fly would be able to track either figures or wide-field motion (but not both) without necessarily relying on the efference copy.

As opposed to (or in conjunction with) efference copy mechanisms, the sensory filters of visual neurons combined with the spatial tuning characteristics we disclose may mediate the control of head and wing movements during active visual behavior. The general conceptual framework, posited by Egelhaaf (Egelhaaf, 1985), is that the receptive field properties and response dynamics can essentially filter figure motion and wide-field motion independently. The key prerequisites are manifest in figure- and wide-field-specific encoding properties by separate identified neurons. In addition to the canonical wide-field HS and VS LPTCs, other lobula plate circuits encode small-object motion and play an important role in figure–ground discrimination. Figure-detecting (FD) cells in larger flies have large receptive fields but are selective for small-object motion (Egelhaaf, 1985; Liang et al., 2012), but the role of such cells in guiding head movements is not as easily predicted from our behavioral data. Do the figure-tracking head responses seen here (Fig. 5B) require input from FD cells? Further experiments will be necessary to determine the nature of the inputs of figure-detecting and wide-field LPTCs on neck motoneurons.

### Mechanosensory influence on head stabilization

Other insects tracking figures on a background of self-generated wide-field motion, such as foraging dragonflies, will follow moving figures with their heads and then steer their wings to intercept them (Olberg et al., 2007). Why are the head-angle responses of tethered fruit flies to simultaneous figure and wide-field stimuli seemingly different from those of dragonflies? One possible answer is that figure tracking with the head, as seen in dragonflies but not in tethered *D. melanogaster*, relies on mechanosensory or other proprioceptive input. Freely flying animals will be able to detect their own movement through various proprioceptive or mechanosensory organs; the flies, in particular, receive inputs to the neck motoneurons from the gyroscopic halteres (Chan and Dickinson, 1996; Huston and Krapp, 2009). It is possible that freely flying flies may not show the large responses to wide-field motion seen here in tethered flies, because the integration of gyroscopic input from halteres could influence the head's position such that tracking of self-induced optic flow is suppressed to allow tracking of figures with the head. In our experiments, the halteres are free to move and are beating along with the wings, and thus the spiking responses of the subset of neck motoneurons that spike only with simultaneous haltere and visual inputs (Huston and Krapp, 2009) are presumably intact. However, the addition of gyroscopic information from body rotations in free flight is likely to inform head movements and adjust the response to visual stimuli. Future experiments involving the mechanosensory system will be necessary to determine the haltere's influence on visually mediated head movements.

### Head-movement behavior may reflect behavioral and ecological demands

A second reason that fruit fly head responses to the compound figure and wide-field stimulus are distinct from figure-tracking head movements of preying dragonflies is that the animals are solving fundamentally different problems. It is well established that fruit flies are able to track figures both while walking (Schuster et al., 2002; Robie et al., 2010) and in tethered flight (Aptekar et al., 2012). However, their ecology does not require them to intercept small moving targets for the purposes of predatory feeding (as with dragonflies or robberflies) or aerial mating pursuits [as with houseflies or blowflies (Land and Collett, 1974)]. The absence of this demand is perhaps the functional reason that fruit flies lack the acute zone found in many chasing flies (Land and Eckert, 1985), and the absence of this zone may be related to fruit flies' failure to aim their heads at small-field figures. We would not be surprised if a fly possessing a distinct acute zone, such as *Coenosia* (Gonzalez-Bellido et al., 2011), showed very different head movements. Without a zone of high acuity on the retina, there is no reason for fruit flies to aim their heads towards a small target. Rather, fruit flies are highly adept at stabilizing self-induced optic flow by following the wide-field motion with their heads, thus enhancing the relative motion of small visual features. This simple gaze-stabilization strategy has been previously reported in larger flies in the context of collision avoidance (Schilstra and van Hateren, 1998; van Hateren and Schilstra, 1999); however, we show here that it persists while a moving figure is not only present, but is actively being tracked by the fly's wings.

## MATERIALS AND METHODS

### Fly preparation and flight simulation arena

**Fig. 6.**
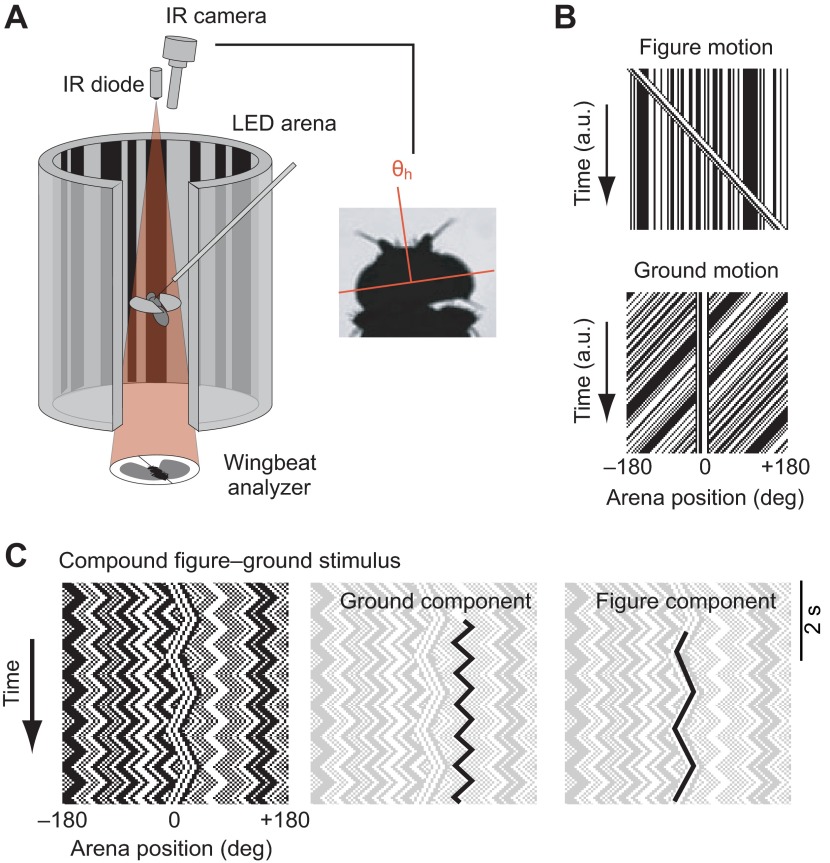
**Experimental setup and visual stimuli.** (A) Flies are rigidly tethered to pins and suspended between an infrared (IR) LED and two photodiodes, which measure the difference between the fly's left and right wingbeat amplitudes (ΔWBA). They are surrounded by an arena of green LED panels on which various stimuli can be displayed. A camera above the fly records an image of the fly's head (inset), and the angle of the head (θ_h_) is measured with tracking software. (B) Our stimulus consisted of a randomly textured wide-field panorama and a randomly textured bar that could be moved independently. Here, we show space–time plots of a stimulus consisting of simplified motion of only one stimulus component. (C) Triangle-wave stimuli showing figure and wide-field motion moving with two different frequencies of oscillation.

Adult female *D. melanogaster*, 3–5 days post-eclosion, were reared from a colony of 200 wild-caught female flies (Card and Dickinson, 2008). Flies were prepared as described previously (Duistermars et al., 2007) by tethering cold-anesthetized flies to tungsten pins. In some flies, we fixed the heads in place using a drop of UV glue at the back of the head, leaving the ocelli unoccluded. Flies were placed in the center of a 32×96 pixel cylindrical LED flight arena (Fig. 6A), also described previously (Reiser and Dickinson, 2010). Each pixel subtended 3.75 deg on the eye, which is less than the 5 deg inter-ommatidial angle (Buchner, 1984). An infrared (IR) LED illuminated the beating wings on an optical sensor (JFI Electronics, Chicago, IL, USA) that detected the amplitude of the left and right

wingbeats. The difference in amplitude between the left and right wings, as processed by this instrument, is proportional to the fly's yaw torque (Tammero et al., 2004). These values were digitized at 1000 Hz [National Instruments data acquisition (NIDAQ) PCI card, Austin, TX, USA] and recorded using MATLAB (The MathWorks, Natick, MA, USA).

### Visual stimuli

We generated two visual stimuli. A salient visual figure consisted of a vertical bar, 30 deg subtended upon the eye, extending from −60 to 60 deg elevation (the full extent of the display). Both the bar and the remaining wide-field ground panorama were composed of a random pattern of vertical 3.75 deg (one display pixel) stripes that were bandpass filtered to ensure that most solid bright or dark elements were 2–4 pixels (7.5–15 deg) in width and that the average contrast of both bar and wide-field panorama was matched at 50% (i.e. half of the display stripes were dark and half were bright; Fig. 6A). The figure occupies only a small field of view (30 deg), whereas the background occupies a wide field of view (the remaining 330 deg). In one set of experiments, we modified the size of the figure from 15 to 180 deg to investigate the influence of figure size on the wing and head responses. In keeping with historical terminology, we shall refer to ‘figure’ and ‘ground’ for the small-field object and wide-field motion stimuli, respectively. The motion of the figure and ground were modulated separately (Fig. 6B) so that they could be controlled in open-loop conditions by a prescribed function (Fig. 6C), or under closed-loop feedback by the time-varying amplitude difference between the two wings. By constructing the stimulus in this way, the only feature distinguishing the bar from the panorama was relative motion.

### Head tracking

An IR-sensitive Basler A601f camera with a 94 mm zoom lens (Edmund Optics, Barrington, NJ, USA) was mounted on a micromanipulator above the arena (Fig. 6A). An IR LED was placed below the fly and images were captured using Motmot, open-source software for video acquisition (Straw and Dickinson, 2009). The frame rate was controlled by a 5 V pulse from a waveform generator (Hewlett-Packard 33120A, Palo Alto, CA, USA), and this 5 V pulse was recorded using the NIDAQ board (described above) for frame synchronization. Using FlyTrax (Pasadena, CA, USA), a Python plugin to Motmot for real-time tracking of a single point in the frame, we tracked the position of a point on the fly's head near the base of one antenna. Video acquisition and head-tracking software were run on an Ubuntu 10.04 LTS platform (Canonical Group Ltd, London, UK) on a Dell PC. To minimize any potential error due to tracking bias, the left side of the head was tracked in half of the trials, and the right side in the other half. Data from Flytrax were imported into MATLAB and analyzed using custom software. We calculated the head yaw angle (Fig. 6A) during each frame and aligned these data with the wingbeat data using the synchronizing pulse that triggered the camera shutter. In this way, we captured the head yaw angle, as well as the two wingbeat amplitude signals and the position of the stimulus pattern, at each frame. Head angle was measured from video collected at 50 frames s^−1^. We found no difference between wing STAFs collected at 1000 Hz and downsampled to those collected at 50 Hz. Because wing kinematics are capable of changing at wingbeat frequency (>200 Hz), and yet STAFs are sufficiently captured at 50 Hz, we feel confident that 50 frames s^−1^ is a sufficient speed to capture the temporal dynamics of head motions. During stimulus presentation (constrained to visual yaw), the flies' heads were generally stable in the roll and pitch axes, as judged by visual inspection of the video sequences.

### Measuring impulse responses to figure and ground motion: experiments

A randomly textured bar figure was stepped incrementally in single pixels according to a seventh-order m-sequence, a pseudorandom pattern of binary digits (−1 or 1) (Ringach and Shapley, 2004; Theobald et al., 2010). The position of the ground was stepped by a second m-sequence, chosen to be maximally uncorrelated with the bar's m-sequence, creating a scenario in which the figure and ground moved randomly and independently. The resulting apparent motion signal is then a series of steps in image position, corresponding to impulses in image velocity. To examine responses to features in various parts of the retinal field, we specified 24 starting positions for the figure stimulus, evenly spaced at 15 deg intervals around the arena. For each trial, the bar was placed in a random starting position and the m-sequences were repeated three times for a total stimulus time of 15.6 s. For each of the 24 start positions, evenly spaced within the 96 pixel azimuth of the LED array, flies flew two trials, one with the original figure m-sequence and one with the figure m-sequence inverted [to remove any residual effects of correlation between the two m-sequences (see Aptekar et al., 2012)], presented in random order. Each trial was interleaved with 5 s of active bar fixation, as described above. Total experiment duration was ~28 min for each fly.

### Measuring impulse responses to figure and wide-field motion: kernel calculation and STAF construction

To measure the linear kernels that describe impulse responses to motion of the figure, the cross-correlation of the difference between the left and right wingbeat amplitudes (ΔWBA) or head yaw angle with the figure stimulus m-sequence was calculated over a sliding window of 127 samples (Theobald et al., 2010). The resulting impulse response estimates were divided by the magnitude of image displacement at each element of the m-sequence (3.75 deg) to give velocity impulse response estimates with dimension ΔWBA(V)/deg for wing steering, or a dimensionless gain estimate (degrees of head movement/degrees of visual stimulus movement) for head movements.

By concatenating the kernels calculated during each trial to the overall estimate for each figure position, we constructed a smooth and robust estimate of the impulse response to figure motion at each of the azimuthal starting positions. The averaged temporal kernels from each spatial position were then plotted along the figure position axis to produce an estimate of the figure STAF. Thus, each column of the figure STAF represents the temporal response kernel for random figure steps centered at that azimuthal location. The figure STAF is therefore a spatiotemporal representation of the fly's object-fixation behavior.

To calculate kernels representing impulse responses to ground motion and thus quantify the fly's optomotor behavior, we cross-correlated the same ΔWBA or head angle response with the ground stimulus m-sequence and constructed the STAF in the same way. The ground STAF is thus parameterized by the position of the figure stimulus; e.g. the kernel at the midline of the ground STAF describes the fly's response to ground motion when the figure stimulus is located in the front and center of the visual field. Each STAF is therefore a three-dimensional surface, with figure position and time on two of the axes and the amplitude of the impulse response on the third. Here, we show these surfaces as heat maps, with warm colors representing large positive correlations and cool colors representing negative correlations. Azimuthal figure position is on the horizontal axis and time is on the vertical axis.
